# Effect of altrenogest treatment before weaning on reproductive performance and production efficiency in primiparous and multiparous sows

**DOI:** 10.1186/s40813-024-00377-7

**Published:** 2024-07-06

**Authors:** Kexiong Liu, Xiaoling Xu, Yuqing Song, Linli Xiao, Junhui Wen, Haiquan Ding, Shuxin Zhao, Dongliang Qiao, Bo Zhang, Aihua Niu, Jiahua Bai, Yan Liu

**Affiliations:** 1 Institute of Animal Husbandry and Veterinary Medicine, Beijing Academy of Agriculture and Forestry Sciences, Beijing, 100097 China; 2 Development Center of Science and Technology, Ministry of Agriculture and Rural Affairs, Beijing, 100176 China; 3 Beijing Zhongyu Pig Breeding Co., Ltd, Beijing, 100194 China

**Keywords:** Altrenogest, Estrus concentration, Follicle development, Lactation, Weaning sows

## Abstract

**Background:**

Most sows will experience negative energy balance during lactation resulting in impaired follicular development. This study aimed to treat 28-day lactating sows with altrenogest (ALT) to suppress follicle enlargement during lactation, and to assess the estrus and reproductive performance post-weaning.

**Methods:**

In this study, we conducted two trials. In trial 1, we monitored the follicular development of lactating sows including 10 primiparous sows and 10 multiparous sows during the whole lactation to confirm the ALT administration time. In trial 2, a total of 42 primiparous and 111 multiparous sows were allocated to three treatments: Ctrl (control group, *n* = 51): no treatment; TAI (timed artificial insemination group, *n* = 51): sows were injected with equine chorionic gonadotropin (eCG) after weaning 24 h and gonadotropin-releasing hormone (GnRH) when they expressed estrus; and AT-TAI (ALT treatment-timed artificial insemination group, *n* = 51): base on the process of TAI group, the sows were fed with 20 mg ALT per day before weaning 10 days. All sows were artificially inseminated twice at 12 h and 36 h after estrus. The follicle size changes and serum hormone levels were explored in this process.

**Results:**

Although the follicle size of multiparous sows was larger than primiparous sows during the whole lactation (*P* < 0.05), similar change trends of follicle size were observed in primiparous and multiparous sows. Meanwhile, the FSH, LH and E_2_ levels of multiparous sows were higher than primiparous sows. The ALT treatment significantly inhibits the increase in follicle size (*P* < 0.05) and reduces the serum levels of FSH, LH and E_2_ (*P* > 0.05). Additionally, ALT treatment increases estrus concentration and the preovulatory follicle size (*P* < 0.05), meanwhile, it delays the weaning-to-estrus interval (WEI, *P* < 0.001). However, the estrus rate, pregnancy rate, total pigs born and born alive did not differ between treatments (*P* > 0.05).

**Conclusions:**

There were significant differences in the size of follicles in the lactation between primiparous and multiparous sows. ALT treatment during the last ten days of lactation concentrated estrus expression leading to higher work efficiency of breeder in batch production, however, with no improvement in reproductive performance.

## Background

In pig production, the weaning-to-estrus interval (WEI), the major component of non-productive days, may be influenced by parity, lactation length, litter size, nutrition, boar exposure, diseases and management [1]. With the genetic improvement and changes in pig management models, the WEI in sows has been shortened from 11.5 to 20.5 days in the 1980s to the current 5–7 days 36, 37. During lactation, since the secretion of gonadotropin-releasing hormone (GnRH) and luteinizing hormone (LH) were suppressed by piglet sucking and negative energy balance (NEB), resulting in the follicle diameter of lactating sows were not exceed 5–6 mm, therefore lactating sows are rarely in estrus and have low ovarian activity [2, 3]. After weaning, the inhibition of GnRH and LH secretion was lifted with the separation of piglets and sows, promoting follicle development and sows estrus [4].

Although the levels of gonadotropins are low during lactation, the growth of follicles is not completely inhibited [3, 5], resulting in differences in the follicle diameter at weaning. Consequently, the WEI of weaning sows varied greatly, although most sows enter estrus within 7 days of weaning [6]. In addition, many factors, including season, litter size and nutrition, affect the follicle development of lactating sows, which leads to greater differences in the follicle diameter [7], especially in the third week after parturition [8]. Prolonged WEI results in elevated culling rates,higher costs [9] and reduced litter size [10]. Specifically, sows entering estrus 3–5 days after weaning exhibit improved reproductive performance compared to those entering estrus < 3 days or 7–10 days after weaning [11]. Additionally, prolonged or dispersed WEI increases the manual labor force including multiple checks and insemination [12].

In the batch production process of pigs, replacement gilts can achieve simultaneous estrus by administering ALT [13]. Many studies have attempted to decrease the impact of negative energy balance on follicular development to improve reproductive performance in subsequent cycles by treating lactating sows with ALT. It has been found that the administration of ALT can increase the size of follicles at ovulation [14] and litter size in sows [15, 16]. However, a recent report suggests that ALT treatment during the last week of 21-day lactation postponed estrus expression after weaning, larger follicle and CL sizes but with no improvement in reproductive performance [17]. Nonetheless, the contribution of ALT on estrus concentration and reproductive performance in weaning sows with 28-day lactation remains unclear.

This study was the first to investigate follicle development in sows throughout lactation. Subsequently, we attempted to increase estrus concentration by administering ALT to sows before weaning. Throughout this process, follicle development, ovulation, and reproductive hormone levels were monitored and measured to establish a foundation for optimizing TAI procedures in weaned sows.

## Methods

Unless expressly specified, all pharmaceutical products utilized in this investigation were procured from Ningbo Sansheng Pharmaceutical (Zhejiang, China).

### Animals and animal management

A total of 173 Landrace×Large White crossbred sows (Primiparous: ~150 kg; Multiparous: ~250 kg) were used in this study. The sows were transferred into the farrowing house at 113 days of gestation. They were housed in farrowing crates (2.10 × 0.70 m) to reach ad libitum feeding 1.8 kg/day (3.35 metabolizable energy (ME), Mcal /kg, 17.9% crude protein, and 1.08% digestible lysine) and water from when they were moved in. After weaning, sows were housed in individual gestation crates (2.10 × 0.65 m) with ad libitum access to water and corn–soybean meal gestation diet (3.35 Mcal ME/kg, 15% of crude protein, and 0.9% of digestible lysine). From the first artificial insemination (AI) until day 30 of gestation, sows were daily fed 3.6 kg. From day 31 until day 90 of gestation, sows were fed 2.2 kg/day. From day 90 until transferred into the farrowing house, sows received 4.2 kg/day. The sows had free access to water and were treated with the same feed (Table 1).

**Table 1:** Ingredients, chemical composition, and nutritional value of the experimental diets for the pig lines

Item	Lactating sows		Weaning sows
**Ingredient**	Appending proportion (%)		
fish meal	3	-	
Soybean hulls	-	6.96	
2% Premixed feed	2	2	
Wheat bran	3	11.6	
Corn grain	56.01	47.11	
Soybean oil	2	0.5	
Soybean meal	18.83	11.8	
Stone powder	1.32	1.21	
*CaHPO*_*4*_	0.84	1.32	
Wheat middling	5	12.5	
Beet meal granules	-	5	
Flour	8	-	
Total	100	100	
**Nutritive index**			
Crude protein (%)	17.64	13.97	
Net energy (Kcal/kg)	-	-	
Digestible energy (Kcal/kg)	3452	3135	
Crude fat (%)	4.98	3.23	
Crude ash (%)	6.1	6.82	
Crude fibers (%)	2.98	6.31	
Ca (%)	0.85	0.85	
Lysine (%)	1.13	0.66	
Vitamin A (IU/kg)	6996	11,872	
Vitamin D3 (IU/kg)	2400	3200	
Vitamin E (mg/kg)	84	96.49	
Vitamin K3 (mg/kg)	7.8	7.8	
Vitamin B12 (mg/kg)	60	40	
Vitamin B1 (mg/kg)	1.8	1.8	
Vitamin B2 (mg/kg)	6.4	6.4	
Vitamin B6 (mg/kg)	3.33	3.33	
Pantothenic acid (mg/kg)	21.78	21.78	
Niacin (mg/kg)	35.64	35.64	
Choline chloride (mg/kg)	249.6	585	
Folacin (mg/kg)	4.36	4,36	
Biotin (mg/kg)	0.44	0.44	
Mn (mg/kg)	29.58	36.7	
Fe (mg/kg)	265	288	
Zn (mg/kg)	79.75	92.5	
Cu (mg/kg)	17.8	22.24	
I (mg/kg)	0.1	0.11	
Se (mg/kg)	0.44	0.46	

### Experimental design

Trial 1.

To explore the follicular development of lactating sows and confirm the ALT feed time, we monitored the follicle size of lactating sows every other day from the first day after farrowing (D0). In addition, the serum was collected every three days from the farrowing day. To avoid the physiological differences caused by parity, 10 primiparous and 10 multiparous sows were used in this trial.

Trial 2.

**Fig. 1:**
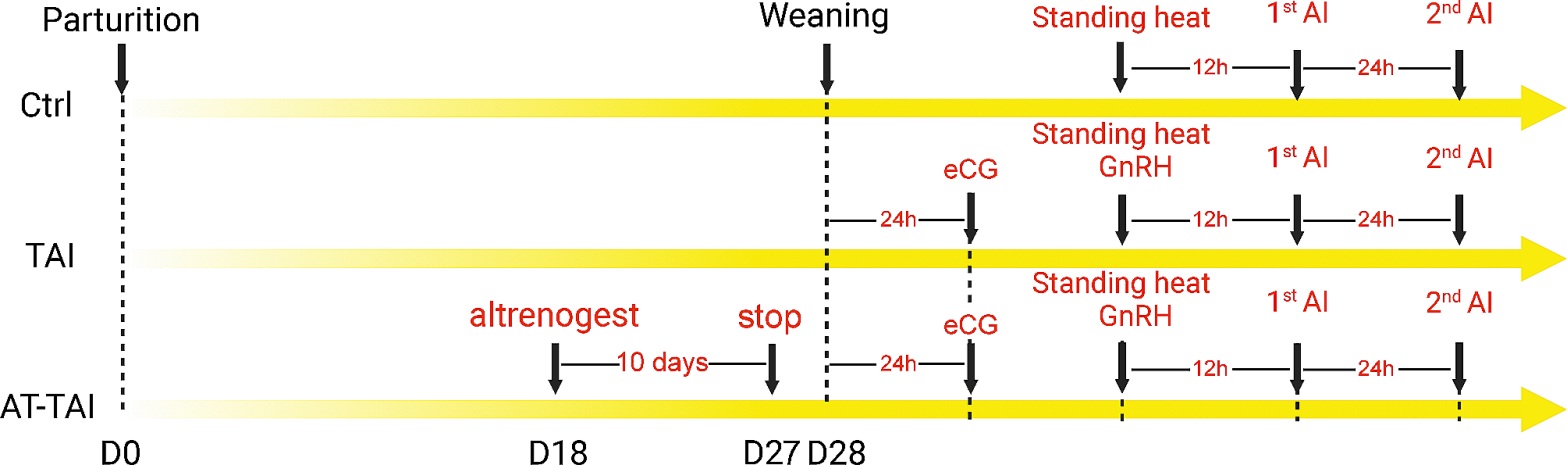
Schematic of experimental design with weaning sows in trial 2

According to the results of trial 1, the trial 2 design and program are shown in Fig. 1. The farrowing sows were randomly divided into three groups (shown in Table 2): control group (Ctrl, *n* = 51), timed artificial insemination group (TAI, *n* = 51), and ALT treatment-timed artificial insemination group (AT-TAI, *n* = 51). The Ctrl group sows received no treatment during lactation and after weaning. The TAI group sows were treated as described in our previous study [12]. In brief, the sows were injected with 1000 IU eCG at 24 h after weaning and 100 ug GnRH when sows showed oestrus. The AT-TAI group sows were orally administered ALT (20 mg per sow per day) from D18 to D27 (total 10 days, farrowing day was considered D0), besides, these sows were given 1000 IU eCG at 24 h after weaning and 100 ug GnRH when sows showed oestrus. The sows were inseminated for the first time 12 h after oestrus and then again 24 h later. The parity and litter size of test sows are shown in Table 2.

**Table 2:** The parity and litter size of test sows

Characteristics	Weaning sows			SEM	*P*-Value
	Ctrl	TAI	AT-TAI		
Parities	51	51	51		
1	13 (25.49%)	15 (29.41%)	14 (27.45%)	-	ns
2	5 (9.80%)	4 (7.84%)	5 (9.80%)		
3	6 (11.76%)	7 (13.73%)	7 (13.73%)		
4	7 (13.73%)	4 (7.84%)	3 (5.88%)		
5	10 (19.61%)	12 (23.53%)	11 (21.57%)		
6	10 (19.61%)	9 (17.65%)	11 (21.57%)		
Total pigs born/litter (mean)	13.47	13.41	12.37	0.275	0.186
Pigs born alive/litter (mean)	10.96	11.16	11.08	0.161	0.883

ns: no significant

### Ultrasound analysis

In trial 1, 10 primiparous and 10 multiparous sows were utilized for assessing follicular growth during lactation. B-ultrasound evaluation of the ovaries was conducted every other day (0800 h) using a Honda HS-1600 V device (Honda Electronics, Tokyo, Japan) with a 5.0-MHz sectorial probe from D1 until D27. In trial 2, ultrasonography assessment was carried out every other day (0800 h) from D19 to D27 during the treatment period, and twice daily (0800 and 2000 h) from oestrus to ovulation. The size of the three largest follicles was recorded. The occurrence of ovulation was affirmed when the count of prominent follicles exhibited a significant decrease compared to the prior scanning session. The follicle development curves during lactation and administration were then plotted.

### Blood collection and hormone analysis

In trial 1, blood samples were collected randomly from 6 primiparous sows and 6 multiparous sows for serum hormone analysis (including E_2_, FSH and LH). From D0 to D28, blood was collected from the caudal vein every three days (0800 h, D0-D28) using tubes with clotting activator. In trial 2, 18 sows from three groups (*n* = 6) were used for blood samples during treatment (0800 h, D21-D28). Following collection, the blood samples were centrifugated at 1,500 g for 8 min. The serum was stored at − 20 °C until analysis. The E_2_, FSH and LH levels were measured by radioimmunoassay (RIA) using commercial RIA kits (Beijing North Institute of Biological Technology, China).

### Estrus detection and artificially inseminate (AI)

Estrus detection was conducted twice daily in the presence of a sexually mature boar, commencing on the day of weaning. This involved a technician using fence-line boar contact with the backpressure test and observing sows for the “standing reflex” when a boar passes by. The estrus time of each sow was recorded. The sows were post-cervically inseminated with pooled semen doses with 1.5 × 10^9^ sperm cells at 12 and 36 h after estrus (maximum three AI). Sows not exhibiting estrus signs until 10 days post-weaning were considered in anestrus.

### Statistical analysis

Statistical analysis was performed using the SPSS software version 24.0 (IBM Corp., Armonk, NY, USA), and the results are presented as means ± SEM. In trial 1, the student’s two-tailed t-test was used to analyze the same-day follicle size and hormone levels of sows with varying parity. In trial 2, each sow was considered as an individual experimental unit. Initially, all models included treatment, parity and previous litter condition as fixed effects. However, no differences were observed in parity, total pigs born, and pigs born alive among the three groups. Consequently, all sows were collectively analyzed with only treatment considered as a fixed effect. The same-day follicle size, hormone levels, WEI, preovulation follicle size, total pigs born and pigs born alive were analyzed by One-way ANOVA, followed by LSD post-hoc test. The estrus, pregnancy and farrowing rates were analyzed and compared using Chi-square tests. *P* < 0.05 were considered statistically significant.

## Results

### Follicular development and hormone levels in sows throughout lactation (trial 1)

**Fig. 2:**
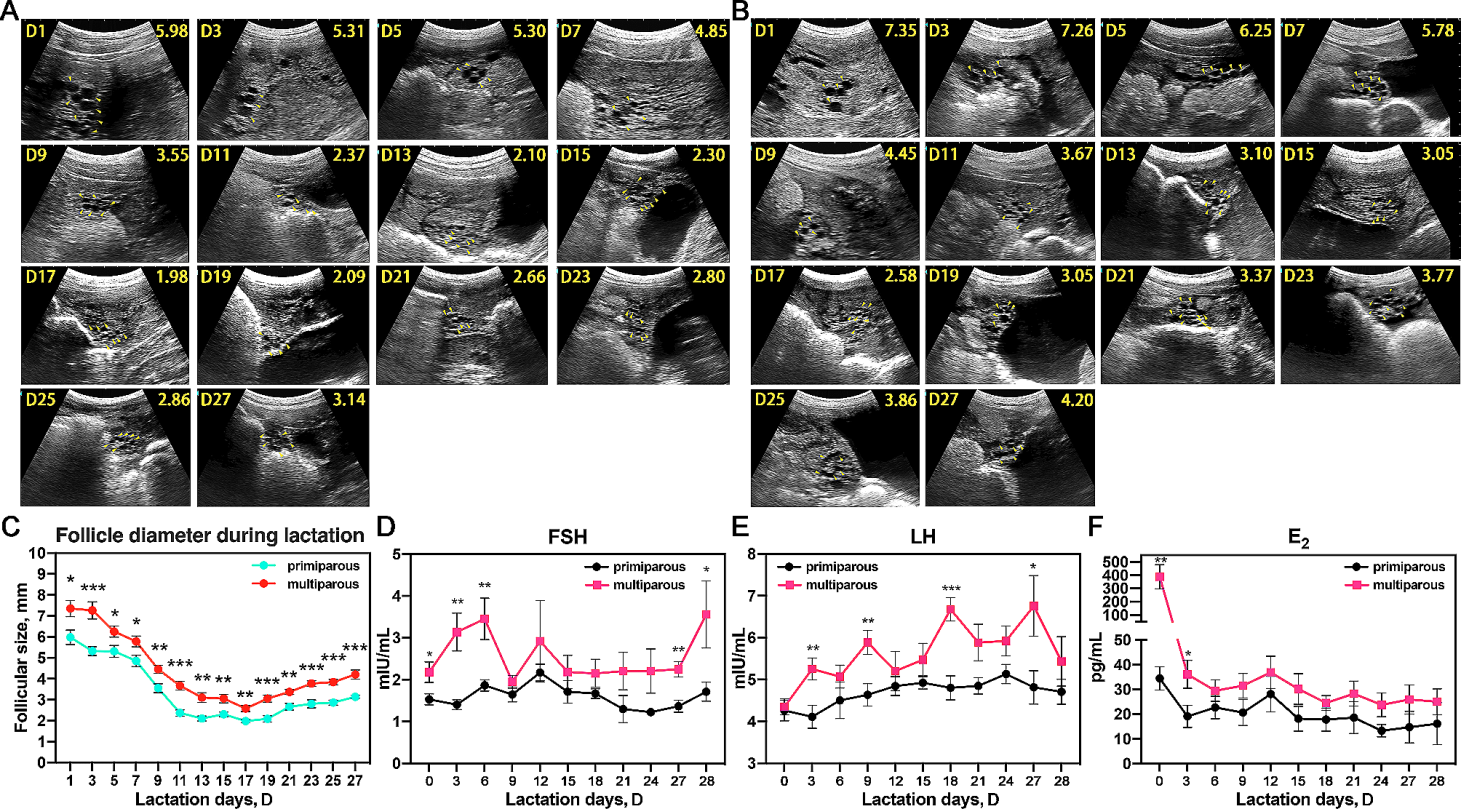
Follicular development and hormone levels of sows during lactation. Representative images of lactation ovary B-ultrasonography of primiparous sows (*n* = 10) (**A**) and multiparous sows (*n* = 10) (**B**). The yellow triangles point to the follicles. The upper left corners of each image represent the time and the upper right corners represent the average follicle diameter on that day. (**C**) The changing curve of sows follicle size during lactation. Only the daily size of primiparous and multiparous sows was compared. The changing curves of sows serum hormone FSH (**D**), LH (**E**) and E_2_ (**F**) during lactation (*n* = 6). * *P* < 0.05, ** *P* < 0.01, *** *P* < 0.001

We initially conducted follicular diameter monitoring of lactating sows, differentiating between primiparous and multiparous sows, due to their physiological variances (Fig. 2A and B). The B-ultrasound images were transformed into follicle developmental curves, revealing that while similar trends of follicular development were observed, the follicle size of multiparous sows was significantly larger than that of primiparous sows at any stage of lactation (Fig. 2C). In addition, the smallest follicle diameter of both primiparous and multiparous sows was observed at D17 and began to increase at D19. Subsequently, we conducted an assessment of reproductive hormone levels. Apart from the E_2_ peak after farrowing, the fluctuations in FSH and LH generally exhibited stability; however, three hormones in multiparous sows were higher than those in primiparous sows, which correlates with their follicle size during lactation (Fig. 2D-F).

### Follicular development and hormone levels of sows during ALT treatment (trial 2)

**Fig. 3:**
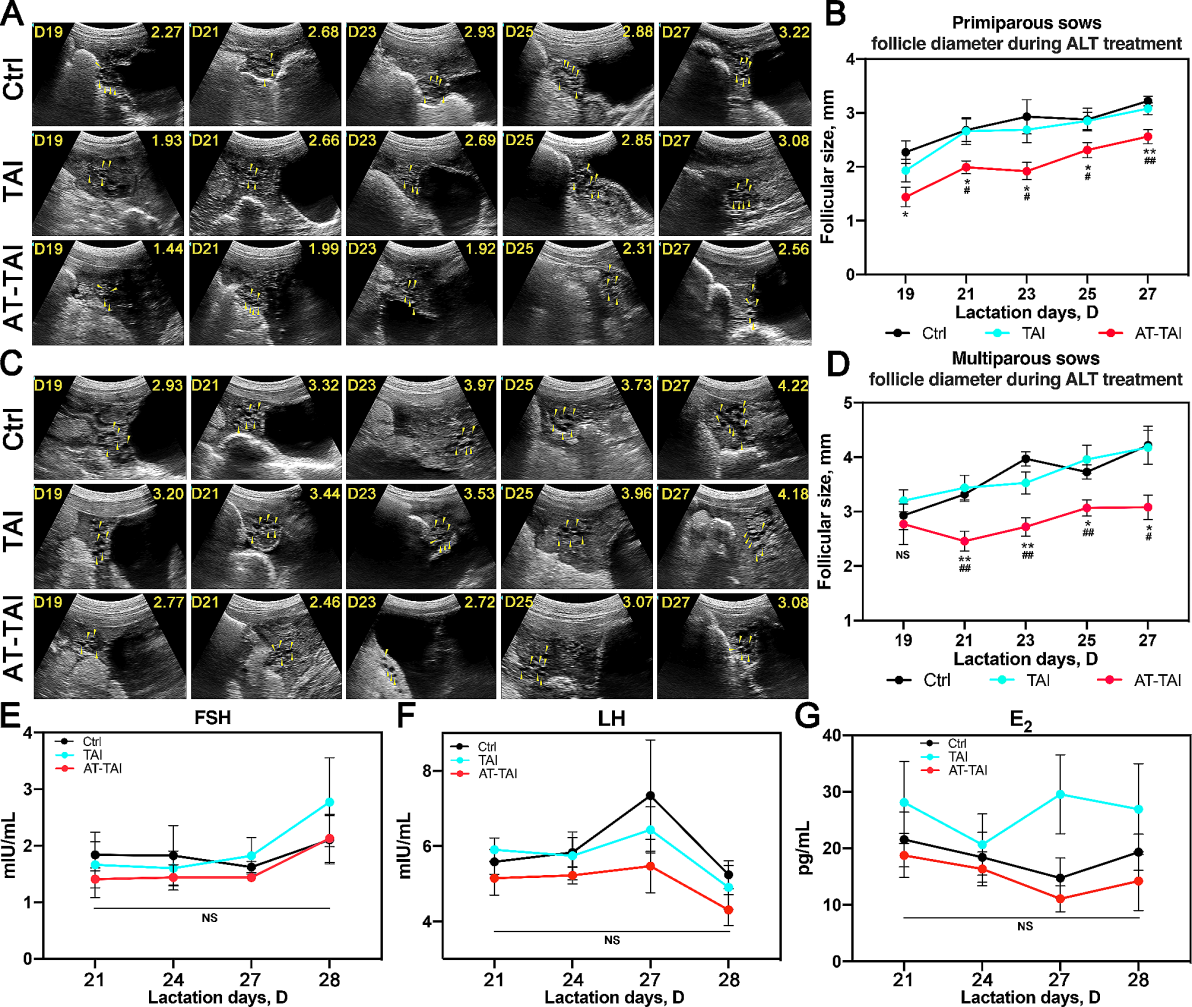
Follicular development and hormone levels of sows during ALT treatment. Representative images of lactation ovary B-ultrasonography of primiparous sows (**A**) and changing curves (**B**) during ALT treatment (*n* = 5). Representative images of lactation ovary B-ultrasonography of multiparous sows (**C**) and changing curves (**D**) during ALT treatment (*n* = 5). The yellow triangles point to the follicles. The upper left corners of each image represent the time and the upper right corners represent the average follicle diameter on that day. The changing curve of sows serum hormone FSH (**E**), LH (**F**) and E_2_ (**G**) during ALT treatment (*n* = 6), * *P*< 0.05, ** *P*< 0.01 (AT-TAI versus Ctrl); ^**#**^*P* < 0.05, ^**##**^*P*< 0.01 (AT-TAI versus TAI); NS: no significant

We chose to feed ALT to sows from D18 to D27 (a total of 10 days) to inhibit follicular enlargement during the NEB period and enhance the synchronization of follicular development after weaning, based on the findings from experiment 1. The ovaries were monitored using B-ultrasound during the ALT treatment (Fig. 3A and C). The results indicated that ALT effectively suppressed follicle growth before weaning in primiparous and multiparous sows, with the follicle diameter in the AT-TAI group being significantly smaller than that in the other two groups (Fig. 3B and D). Correspondingly, FSH, LH, and E_2_ levels in the AT-ALT group were lower compared to those in the other two groups; however, no statistically significant differences were observed among the three groups (Fig. 3E-G). Additionally, the follicles in the ALT-treated group were larger than those in the other two groups 12 h before ovulation, regardless of parity (Primi: *P* = 0.036; Multi: *P* = 0.023, Table 3).

**Table 3:** The reproductive performance of weaning sows

Terms	Ctrl	TAI	AT-TAI	SEM	*P*-Value
n	51	51	51	-	-
WEI, h	94.29^a^	87.82^a^	109.64^b^	2.04	< 0.001
Oestrus rate^1^ (%)	68.63^a^ (35/51)	86.27^b^ (44/51)	86.27^b^ (44/51)	-	0.035
Preovulation follicle size (mm)					
Primiparous	5.91^a^	5.62^a^	7.19^b^	0.29	0.036
Multiparous	5.33^a^	6.18^ab^	6.77^b^	0.25	0.023
Pregnancy rate^2^ (%)	91.43 (32/35)	93.18 (41/44)	95.45 (42/44)	-	0.900
Farrowing rate^3^ (%)	96.88 (31/32)	97.56 (40/41)	95.24 (40/42)	-	0.839
Total pigs born/litter (mean)	15.84	15.95	15.33	0.31	0.648
Pigs born alive/litter (mean)	14.19	13.73	13.53	0.25	0.865

^a, b^ Different letters in the same row represent significant differences (*P* < 0.05)

^1^ Oestrus rate = no. of oestrus sows/no. of treated sows

^2^ Pregnancy rate = no. of pregnant sows/no. of oestrus sows

^3^ Farrowing rate = no. of farrowing sows/no. of pregnant sows

### Effect of ALT treatment reproductive performance in weaning sows (trial 2)

**Fig. 4:**
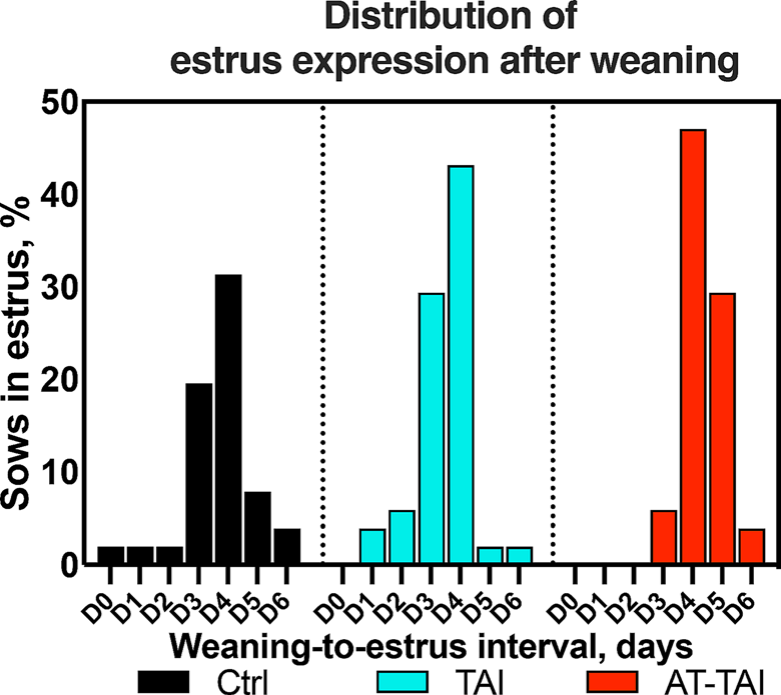
The estrus distribution in three groups after weaning (weaning day was considered D0)

The oestrus rate was significantly higher in the AT-TAI and TAI groups compared to the Ctrl group (*P* = 0.035, Table 3). Additionally, the ALT-treatment sows exhibited a longer WEI than the other two groups (*P* < 0.001, Table 3). As shown in Fig. 4, most sows expressed estrus on D3 and D4 after weaning in Ctrl and TAI groups, while on D4 and D5 in the AT-TAI group (weaning day was considered D0). Notably, no sow showed signs of estrus before D3 in the AT-TAI group, indicating that the ALT treatment reduced the duration of estrus in this herd. However, there were no differences in pregnancy rate, farrowing rate, total number of piglets born and born alive (*P* > 0.05, Table 3).

## Discussion

As far as we know, we have monitored for the first time the follicular development of sows throughout the entire 28-day lactation period, revealing a pattern of initial decline followed by subsequent increase. Subsequently, we began to feed sows with ALT on the day of the smallest follicle diameter until weaning. The findings indicated that while there was no enhancement in the reproductive performance of weaning sows, it led to an increase in estrus concentration among them, thereby enhancing work efficiency and worker welfare.

It is reported that ovarian follicle growth during lactation determines the reproductive performance of weaned sows [8]. In the period of lactation, the existence of piglets, the suckling reflex, and the catabolic state resulting from milk production impede the release of hypothalamic GnRH and, consequently, the pituitary secretion of LH and FSH, preventing ovarian follicles from growing above 5–6 mm in diameter [2]. However, we observed that the follicle diameter was greater than 5 mm during the first week of lactation in both primiparous and multiparous sows, meanwhile, the diameter of sow follicles exhibits a pattern of initial decline followed by subsequent increase. Although our results contradict many studies, we found similar reports [18, 19]. The changes in progesterone and estrogen secretion during the perinatal period, as well as the activity of LH in the postpartum period, may contribute to the presence of large follicles one week after delivery [20]. In addition, we observed that the follicle size of multiparous sows was larger than that of primiparous sows throughout the entire lactation period. Likewise, the serum hormones including FSH, LH and E_2_ in multiparous sows were higher than in primiparous sows, which approve the changes in follicle size during the whole lactation period. We believe that the disparity in body condition during lactation between primiparous and multiparous sows is primarily responsible for this phenomenon. It is reported that undernutrition during lactation often results in NEB, negatively affecting reproductive processes in the primiparous sow in particular [21], including follicle size [22] and reproductive hormone levels [23, 24].

During lactation, follicular growth is not completely inhibited, and the varying length of WEI is partially attributed to the differing follicle sizes in sows during this period [5]. It is reported that variations in the follicles of pig herds usually occur on days 14–21 of lactation [25], meanwhile, the follicle size began to increase at D18 in our B-ultrasound results of the whole lactation. Due to 28 days of lactation, we fed 10 days of ALT before weaning. During these 10 days, we observed the follicle sizes of ALT-treated sows were smaller than those in the other two groups, which indicated that ALT has an inhibitory effect on lactating sows follicular size. We also noted a similar report, in which stagnant enlargement was observed in follicle size during the ALT feeding period [17]. Additionally, although the growth and development of follicles were suppressed, the FSH, LH and E_2_ levels were not significantly lower than the other groups during ALT treatment. The reason may be the presence of natural inhibition, including suckling by the piglets and lactational catabolic state, stimulates the sow to release peptidergic neurotransmitters blocking GnRH secretion and consequently LH secretion [26].

As anticipated, the administration of ALT effectively synchronizes estrus in weaned sows, with a reduced number of sows displaying signs of estrus within three days after weaning. Two similar studies found that 86.2% and 87.5% of primiparous sows treated with ALT for five days post-weaning exhibited estrus within 4–7 and 3–10 days after ALT withdrawal, respectively [27, 28]. In the current study, all sows treated with ALT exhibited estrus within 3–6 days, compared to 76.47% and 62.75% of sows in the TAI and Ctrl groups, respectively. This indicated that ALT increased the estrus concentration of weaning sows, despite resulting in a 0.63 or 0.91-day delay in WEI compared to the Ctrl and TAI groups, which is not expected to impact the number of non-productive days. This is supported by the estrus rate of the AT-TAI group within six days after weaning, which was comparable to the TAI group and higher than the Ctrl group. In addition, a report confirmed this idea that using ALT from four days before weaning to two days after weaning does not affect total estrus within seven days of ALT withdrawal (95.12% vs. 90.00%, respectively) [29]. Therefore, strategically utilizing ALT can synchronize estrus expression without significantly prolonging WEI. Furthermore, a higher number of synchronized sows in estrus supports the implementation of fixed-time artificial insemination (FTAI) to enhance worker productivity and welfare [30, 31].

Afterward, we analyzed the influence of parity on the estrus rate and determined that the lowest estrus rate observed in non-injected primiparous sows was the primary factor contributing to the lowest estrus rate in the Ctrl group (data not shown), consistent with findings from our previous study [12]. We believe that the low estrus rate of primiparous sows is primarily attributed to small follicles at weaning and excessive weight loss during lactation. Our results also suggest that the administration of eCG can effectively stimulate follicular development and promote estrus in primiparous sows. However, treatment with ALT increased the WEI of primiparous sows in the AT-TAI group, leading to scattered estrus. Therefore, we propose that pre-treatment of multiparous sows with ALT alone and administering eCG and GnRH to all weaning sows can achieve a favorable synchronization effect. This is based on our result and the reports from other groups indicating that primiparous sows had small ovarian follicles at weaning [8], and the ALT treatment for primiparous sows would lead to a greater disparity in weaning follicle size between primiparous and multiparous sows, causing scattered estrus in pig herds.

About the litter size, ALT administration during lactation has contradictory effects. It is reported that pre-weaning ALT treatment leads to a 1.8 increase in the total number of piglets born, due to the presence of larger and more uniform follicles, as well as a higher number of healthy viable embryos following fertilization [15]. Whereas, the increase in litter size was not found in another study, consistent with our findings [29]. We think the basic litter size situation is one of the reasons causing the litter size difference. Lopes and Am-In reported a litter size of 12.2 or 8 piglets in the control group, while our study observed a mean litter size of more than 15 piglets [15, 32]. In addition, a recent study found that combining estrus synchronization and superovulation treatments affects fertilization, embryo production, and reproductive tract gene expression [33]. Moreover, sows usually undergo negative energy balance during lactation [2], which is the main cause leading to poor ovulation rate, embryo survival, and reproductive performance [21, 34]. Therefore, the physical recovery of the sow after weaning is crucial to the reproductive performance of the next litter. Some studies indicated that treatment sows with ALT could improve ovulation rate, number of viable embryos, and litter size by giving more time to recover body condition after lactation [16, 35]. Unlike other studies, we stopped ALT before weaning, however, they withdrew ALT post-weaning. Hence, the AT-TAI sows were given no additional recovery time compared with the other two groups. Nevertheless, treated sows with ALT during lactation may enhance the efficiency of the pig farm including shortening the working hours of workers for estrus detection and AI. In a study involving sows with a 21-day lactation, sows were fed ALT one week before weaning and Gianluppi asked: whether feeding ALT before weaning can inhibit estrus in 28-day lactation sows [17]. We believe our findings provide the most comprehensive answer. Furthermore, we will further explore ways to improve reproductive performance based on this result.

## Conclusions

The pattern of follicle development in primiparous and multiparous sows exhibited similarities during lactation, but the follicle size of multiparous sows was larger than primiparous sows consistently. Additionally, ALT treatment during the last ten days of a 28-day lactation concentrated estrus expression post-weaning. However, reproductive performance was of weaning sows not improved by the pre-weaning treatment with ALT.

## Data Availability

No datasets were generated or analysed during the current study.

## Abbreviations

ALT: Altrenogest
FSH: Follicle-Stimulating Hormone
LH: Luteinizing Hormone
E_2_: Estradiol
Ctrl: Control
TAI: Timed Artificial Insemination
AT-TAI: Altrenogest Treatment-Timed Artificial Insemination
WEI: Weaning-To-Estrus Interval
eCG: Equine Chorionic Gonadotropin
GnRH: Gonadotropin-Releasing Hormone
AI: Artificial Insemination
FTAI: Fixed-Time Artificial Insemination
NEB: Negative Energy Balance

## References

[1] Vesseur P, Kemp B, Hartog LA. Causes and Consequences of Variation in Weaning to Oestrus Interval in the Sow. PhD Thesis, 1997;165pp.

[2] Quesnel H (2009). Nutritional and lactational effects on follicular development in the pig. Soc Reprod Fertil Suppl.

[3] Knox RV (2023). Follicle development in pigs: state of the art. Mol Reprod Dev.

[4] Knox RV (2005). Recruitment and selection of ovarian follicles for determination of ovulation rate in the pig. Domest Anim Endocrinol.

[5] Lucy MC, Liu J, Boyd CK, Bracken CJ (2001). Ovarian follicular growth in sows. Reprod Suppl.

[6] Hoshino Y, Koketsu Y (2008). A repeatability assessment of sows mated 4–6 days after weaning in breeding herds. Anim Reprod Sci.

[7] Costermans NGJ, Teerds KJ, Kemp B, Keijer J, Soede NM (2023). Physiological and metabolic aspects of follicular developmental competence as affected by lactational body condition loss. Mol Reprod Dev.

[8] Lopes TP, Padilla L, Bolarin A, Rodriguez-Martinez H, Roca J (2020). Ovarian follicle growth during Lactation determines the Reproductive performance of weaned sows. Animals.

[9] Koketsu Y, Tani S, Iida R (2017). Factors for improving reproductive performance of sows and herd productivity in commercial breeding herds. Porcine Health Manag.

[10] Tummaruk P, Lundeheim N, Einarsson S, Dalin AM (2001). Effect of birth litter size, birth parity number, growth rate, backfat thickness and age at first mating of gilts on their reproductive performance as sows. Anim Reprod Sci.

[11] Poleze E, Bernardi ML, Filha WSA, Wentz I, Bortolozzo FP (2006). Consequences of variation in weaning-to-estrus interval on reproductive performance of swine females. Livest Sci.

[12] Bai JH, Qin YS, Zhang SL, Xu XL, Song YQ, Xiao LL, Feng T, Tian JH, Liu Y (2021). A comparison of the reproductive performance in primiparous sows following two timed artificial insemination protocols. Animal.

[13] Xiao H, Sun P, Sun F, Qiu J, Wang J, Wang J, Lin Y, Gong X, Zhang L, Zhang S, Cao X (2019). Pharmacokinetics of altrenogest in gilts. J Vet Pharmacol Ther.

[14] Van Leeuwen JJ, Williams SI, Kemp B, Soede NM (2010). Post-weaning Altrenogest treatment in primiparous sows; the effect of duration and dosage on follicular development and consequences for early pregnancy. Anim Reprod Sci.

[15] Lopes TP, Bolarín A, Martínez EA, Roca J (2017). Altrenogest treatment before weaning improves litter size in sows. Reprod Domest Anim.

[16] Van Leeuwen JJ, Williams SI, Martens MR, Jourquin J, Driancourt MA, Kemp B, Soede NM (2011). The effect of different postweaning altrenogest treatments of primiparous sows on follicular development, pregnancy rates, and litter sizes. J Anim Sci.

[17] Gianluppi RDF, Lucca MS, Quirino M, Mellagi APG, Ulguim RDR, Bortolozzo FP (2021). Altrenogest treatment during the last week of lactation on ovarian traits and subsequent reproductive performance of primiparous and multiparous sows. Theriogenology.

[18] Zemitis J, Bouwman EG, Langendijk P, Wettere WHEJv, Kirkwood RN (2015). Postpartum injection of human chorionic gonadotrophin: effects on sow ovarian follicles. J Swine Health Prod.

[19] Sesti LA, Britt JH (1994). Secretion of gonadotropins and estimated releasable pools of gonadotropin-releasing hormone and gonadotropins during establishment of suckling-induced inhibition of gonadotropin secretion in the sow. Biol Reprod.

[20] De Rensis F, Hunter MG, Foxcroft GR (1993). Suckling-induced inhibition of luteinizing hormone secretion and follicular development in the early postpartum sow. Biol Reprod.

[21] Schenkel AC, Bernardi ML, Bortolozzo FP, Wentz I (2010). Body reserve mobilization during lactation in first parity sows and its effect on second litter size. Livest Sci.

[22] Costermans NGJ, Keijer J, van Schothorst EM, Kemp B, Keshtkar S, Bunschoten A, Soede NM, Teerds KJ (2019). In ovaries with high or low variation in follicle size, granulosa cells of antral follicles exhibit distinct size-related processes. Mol Hum Reprod.

[23] Kauffold J, Gottschalk J, Schneider F, Beynon N, Wähner M (2008). Effects of feeding level during lactation on FSH and LH secretion patterns, and follicular development in primiparous sows. Reprod Domest Anim.

[24] Quesnel H, Pasquier A, Mounier AM, Prunier A (1998). Influence of feed restriction during lactation on gonadotropic hormones and ovarian development in primiparous sows. J Anim Sci.

[25] Young MG, Tokach MD, Aherne FX, Main RG, Dritz SS, Goodband RD, Nelssen JL (2004). Comparison of three methods of feeding sows in gestation and the subsequent effects on lactation performance. J Anim Sci.

[26] Quesnel H, Prunier A (1995). Endocrine bases of lactational anoestrus in the sow. Reprod Nutr Dev.

[27] Fernández L, Diez C, Ordóñez J, Carbajo M. Reproductive performance in primiparous sows after postweaning treatment with a progestagen. J Swine Health Prod. 2005;13.

[28] Werlang RF, Argenti LE, Fries HC, Bernardi ML, Wentz I, Bortolozzo FP (2011). Effects of breeding at the second oestrus or after post-weaning hormonal treatment with altrenogest on subsequent reproductive performance of primiparous sows. Reprod Domest Anim.

[29] Kitkha S, Boonsoongnern A, Ratanavanichrojn N, Jirawattanapong P, Pinyopummin A (2017). Effects of altrenogest treatment in sows on the variation of piglet birth weight and pre-weaning piglet performance. Agric Nat Resour.

[30] Baroncello E, Bernardi ML, Kummer AD, Wentz I, Bortolozzo FP (2017). Fixed-time post-cervical artificial insemination in weaned sows following buserelin use combined with/without eCG. Reprod Domest Anim.

[31] Knox RV, Willenburg KL, Rodriguez-Zas SL, Greger DL, Hafs HD, Swanson ME (2011). Synchronization of ovulation and fertility in weaned sows treated with intravaginal triptorelin is influenced by timing of administration and follicle size. Theriogenology.

[32] Am-In N, Kirkwood RN (2019). Feeding altrenogest during late lactation improves fertility of primiparous sows nursing smaller litters. Can J Vet Res.

[33] Gonzalez-Ramiro H, Gil MA, Cuello C, Cambra JM, Gonzalez-Plaza A, Vazquez JM, Vazquez JL, Rodriguez-Martinez H, Lucas-Sanchez A, Parrilla I, Martinez CA, Martinez EA (2023). The Use of a brief synchronization treatment after Weaning, combined with Superovulation, has Moderate effects on the Gene expression of surviving Pig blastocysts. Animals.

[34] Zak LJ, Cosgrove JR, Aherne FX, Foxcroft GR (1997). Pattern of feed intake and associated metabolic and endocrine changes differentially affect postweaning fertility in primiparous lactating sows. J Anim Sci.

[35] Patterson J, Wellen A, Hahn M, Pasternak A, Lowe J, DeHaas S, Kraus D, Williams N, Foxcroft G (2008). Responses to delayed estrus after weaning in sows using oral progestagen treatment. J Anim Sci.

[36] Belstra BA, Flowers WL, See MT (2004). Factors affecting temporal relationships between estrus and ovulation in commercial sow farms. Anim Reprod Sci.

[37] Behan JR, Watson PF (2005). The effect of managed boar contact in the post-weaning period on the subsequent fertility and fecundity of sows. Anim Reprod Sci.

